# Determination of Markers of Successful Implementation of Mental Health Apps for Young People: Systematic Review

**DOI:** 10.2196/40347

**Published:** 2022-11-09

**Authors:** Holly Alice Bear, Lara Ayala Nunes, John DeJesus, Shaun Liverpool, Bettina Moltrecht, Lakshmi Neelakantan, Elinor Harriss, Edward Watkins, Mina Fazel

**Affiliations:** 1 Department of Psychiatry University of Oxford Oxford United Kingdom; 2 Department of Psychology York University Toronto, ON Canada; 3 Faculty of Health, Social Care and Medicine Edge Hill University Ormskirk United Kingdom; 4 Research Department for Clinical, Educational and Health Psychology University College London London United Kingdom; 5 Centre for Longitudinal Studies University College London London United Kingdom; 6 Bodleian Health Care Libraries University of Oxford Oxford United Kingdom; 7 School of Psychology University of Exeter Exeter United Kingdom

**Keywords:** adolescent mental health, smartphones, mobile apps, apps, implementation science, mobile phone

## Abstract

**Background:**

Smartphone apps have the potential to address some of the current issues facing service provision for young people’s mental health by improving the scalability of evidence-based mental health interventions. However, very few apps have been successfully implemented, and consensus on implementation measurement is lacking.

**Objective:**

This review aims to determine the proportion of evidence-based mental health and well-being apps that have been successfully adopted and sustained in *real-world* settings. A secondary aim is to establish if key implementation determinants such as coproduction, acceptability, feasibility, appropriateness, and engagement contribute toward successful implementation and longevity.

**Methods:**

Following the PRISMA (Preferred Reporting Items for Systematic Reviews and Meta-Analyses) guidelines, an electronic search of 5 databases in 2021 yielded 18,660 results. After full-text screening, 34 articles met the full eligibility criteria, providing data on 29 smartphone apps studied with individuals aged 15 to 25 years.

**Results:**

Of 34 studies, only 10 (29%) studies were identified that were evaluating the effectiveness of 8 existing, commercially available mental health apps, and the remaining 24 (71%) studies reported the development and evaluation of 21 newly developed apps, of which 43% (9/21) were available, commercially or otherwise (eg, in mental health services), at the time of enquiry. Most studies addressed some implementation components including adoption, acceptability, appropriateness, feasibility, and engagement. Factors including high cost, funding constraints, and lengthy research processes impeded implementation.

**Conclusions:**

Without addressing common implementation drivers, there is considerable redundancy in the translation of mobile mental health research findings into practice. Studies should embed implementation strategies from the outset of the planned research, build collaborations with partners already working in the field (academic and commercial) to capitalize on existing interventions and platforms, and modify and evaluate them for local contexts or target problems and populations.

**Trial Registration:**

PROSPERO CRD42021224365; https://tinyurl.com/4umpn85f

## Introduction

### Background

There has been a proliferation in the number of smartphone apps being developed, both commercially and in academic research programs, which aim to improve mental health and well-being. Recent estimates suggest that anywhere from 10,000 [1] to 22,750 mental health apps exist [2]. Although many of these apps can be accessed directly by individuals in the commercial app marketplace as self-care tools, they are also playing an increasing role in clinical services, supplementing or enhancing traditional interventions [3]. The rapid expansion in the research and development of mental health and well-being apps highlights how much interest and potential there is thought to be in the mobile health arena.

Most common mental health disorders, including depression and anxiety, have their onset during adolescence and, if not successfully resolved, can lead to negative impacts well into adulthood [4,5]. Given the increasing number of young people (between the ages of 15 and 25 years) using digital technologies, smartphone-based interventions provide a scalable solution to support this group to manage their mental health and well-being [6]. Apps have the potential to address some of the accessibility issues in service provision for young people’s mental health, especially for underserved populations. Emerging evidence suggests that some apps may produce significant symptom improvement across multiple outcomes compared with waitlist or control conditions [7-9]. Despite this promise, empirical research often fails to translate into meaningful and sustained implementation in “real-world” settings [10,11]. This can be attributed, in part, to the complex and lengthy process of implementing and maintaining evidence-based approaches in practice, as well as the commercial and regulatory complexities of scaling up mobile technologies in health services [12,13]. Besides innovation and efficacy, other factors, including user engagement, usability, acceptability, accessibility, and low cost, are key prerequisites for adoption, scalability, and uptake [14-17].

Given these multifaceted challenges, it is important to identify what facilitates and inhibits the implementation of mobile mental health interventions [10]. Currently, our understanding of how mental health apps are implemented in real-world settings is limited in several ways. Foremost, implementation processes and outcomes from research trials are seldom recorded or reported, and implementation efforts often lack a solid theoretical or model-based approach, making it difficult to understand and explain how and why implementation succeeds or fails [18,19]. In the context of mobile mental health apps, successful implementation can be measured by the extent to which the intervention has been embedded into service provision, the number of app users, the frequency of app use, app engagement, and evidence of sustained use following the end of a research trial [20,21]. Assessing implementation outcomes using a conceptually grounded framework allows for a systematic assessment of outcomes while also supporting the rigor and reproducibility of implementation research and providing building blocks for the implementation of future interventions.

Existing scoping and systematic reviews have focused on reviewing and critically appraising the methodological rigor and quality of implementation effectiveness studies, reporting implementation outcomes as their primary outcomes [22,23]. To the best of our knowledge, no review has taken a systematic approach to assessing the successful implementation and sustainment of all evidence-based mental health apps for young people. In this review, we assessed the factors influencing implementation success according to a set of implementation outcome criteria based on a modified version of the implementation framework by Proctor et al [24]. In total, 10 implementation variables were examined, of which 8 were from the Proctor model: acceptability (perceived usefulness and satisfaction with a technology), appropriateness (fitness for purpose), feasibility (extent to which a technology was successfully used), fidelity (implementation as intended), cost (financial impact of technology implementation), adoption (technology uptake and use), penetration (spread or reach of the technology), and sustainability (sustained uptake by users or maintenance or integration of a technology within a health care service). Two additional relevant outcomes were added: coproduction (user involvement in intervention development and evaluation) and engagement (adherence and dropout) [25,26].

Although conceptually distinct constructs, the implementation variables listed above are dynamically interrelated and sequentially contingent on one another [24,27]. For an app to be engaging, widely adopted, and well sustained, it must first be acceptable, appropriate, and feasible. It may be that an app-based intervention is deemed highly relevant and applicable to young people’s needs (high appropriateness) but may be costly to download and time intensive (low feasibility). Similarly, an intervention may be considered by a mental health service as a good fit to address young people’s needs (high appropriateness); nevertheless, the service user may be reluctant to use it if they dislike a certain feature of the intervention (low acceptability). Given the potential benefits of smartphone apps in supporting the mental health and well-being of young people, it is critical that researchers and app developers place greater emphasis on enhancing the engagement, implementation, and scalability of efficacious interventions in local contexts or specific populations.

### Research Questions

The aim of this review was to determine how successfully evidence-based mobile apps, which aim to promote well-being and mental health outcomes in young people, are adopted, scaled up, and sustained in real-world settings. The research questions of interest were as follows:

1. What proportion of evidence-based mental health apps are sustained and adopted after development?
2. What components are needed for successful implementation outcomes and what are the common barriers?

## Methods

The systematic review protocol was registered in PROSPERO (CRD42021224365).

### Literature Search and Search Strategy

An information specialist (EH) performed an electronic search of the following databases from January 1, 2011, to the search date on February 2, 2021: Ovid Embase, Ovid MEDLINE, Ovid PsycINFO, Cochrane Database of Systematic Reviews, and Cochrane Central Register of Controlled Trials. The search strategies used text words and relevant indexing to capture the concepts of studies on the effectiveness or trials of mental health apps for young people. The search strategy was guided by similar reviews exploring digital mental health interventions for young people [25], and the terms for apps were derived from Cochrane reviews [28,29]. The full search strategies are available in Multimedia Appendix 1. Duplicates were removed following the method described by Falconer [30], and records were then screened by titles and abstracts to complete the process manually. The reference lists of included studies and relevant systematic reviews were assessed for additional relevant studies. All references were exported to Endnote X9 (Clarivate) and then to the systematic review software Rayyan [31].

### Inclusion and Exclusion Criteria

The focus of this review was the implementation of app-based interventions that aim to promote mental health and well-being, prevent mental health problems, and treat existing mental health problems in young people. Screened articles were included if (1) the study targeted young people with a mean age of 15 to 25 years, with or without a formal mental health or physical health diagnosis (eg, targeting anxiety in adolescents with diabetes); (2) the intervention was an efficacious “native” mobile app (ie, not on a web browser), whose primary aim was to promote well-being, prevent mental health problems, or treat existing mental health problems; (3) the primary outcome was a measure of mental health or well-being, including change in anxiety and depressive symptoms, diagnosis, problem severity, problem improvement, recovery, remission, or more general change in mental health or well-being across at least 2 time points (eg, baseline and after the intervention or follow-up); and (4) the intervention was efficacious, that is, it had beneficial mental health or well-being outcomes compared with any other type of digital intervention, usual care (eg, psychotherapy), waitlist control group, or no-intervention control group. Both randomized and nonrandomized studies were considered for inclusion.

Articles were excluded if (1) the mean age of participants was not 15 to 25 years; (2) the intervention was not a mobile app, such as other digital interventions, including therapy delivered by phone, SMS text message, video platforms, or PC (eg, computer-based cognitive behavioral therapy); (3) the apps used were not efficacious (ie, there was no significant improvement in mental health or well-being compared with a control group); (4) the apps used did not include an intervention component (ie, primarily focused on diagnosis or assessment); (5) the studies did not report mental health outcomes or the primary outcome was physical (eg, blood sugar levels or exercise); and (6) the studies did not have a control group. Gray literature was not included in the search.

### Study Selection

**Figure 1:**
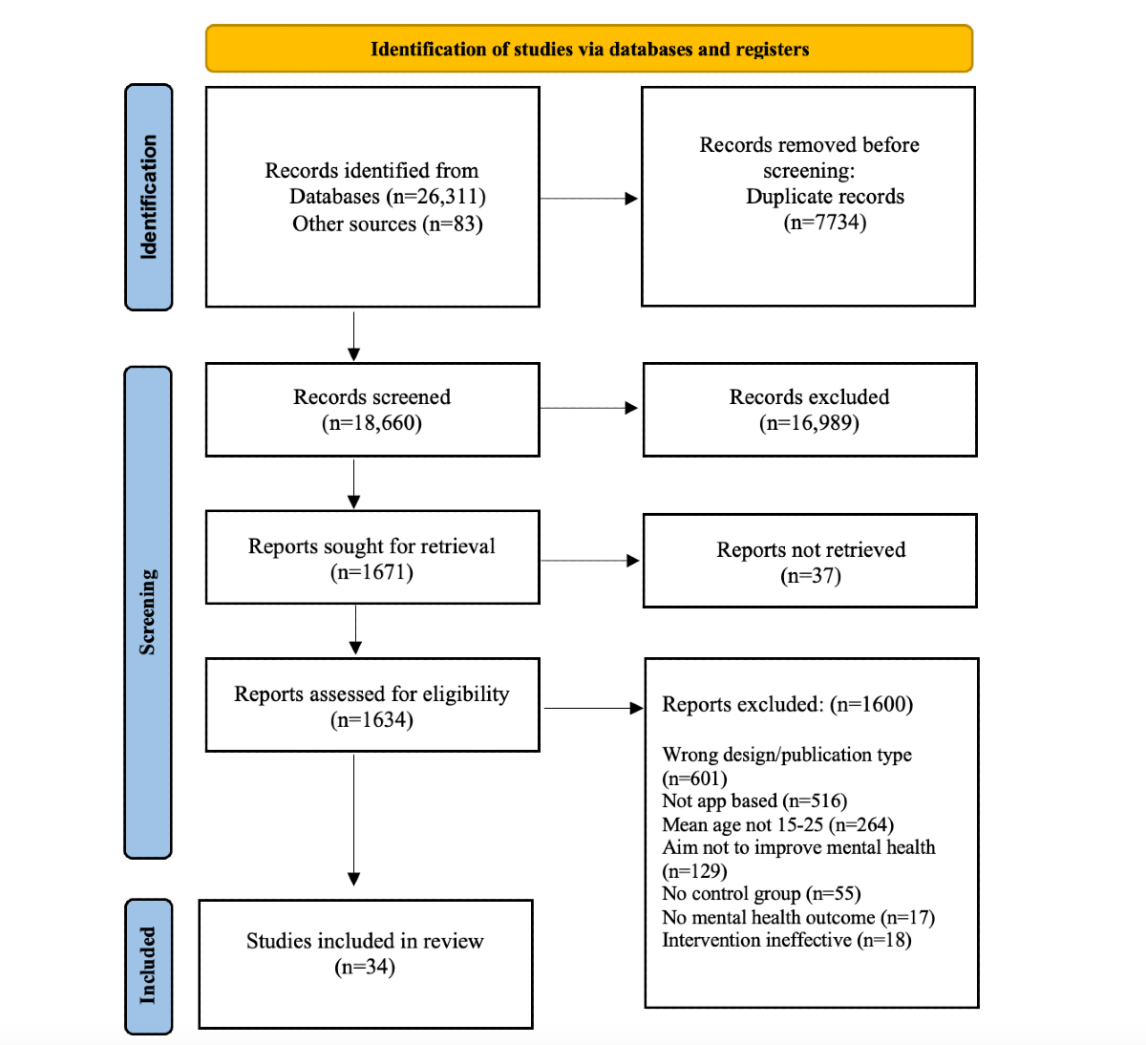
PRISMA (Preferred Reporting Items for Systematic Reviews and Meta-Analyses) flowchart of the study selection process.

In accordance with the PRISMA (Preferred Reporting Items for Systematic Reviews and Meta-Analyses) guidelines [32], the flowchart presented in Figure 1 provides step-by-step details of the study selection procedure. The search strategy identified 18,660 citations after the removal of duplicates. Of these 18,660 studies, 1634 (8.75%) were considered potentially relevant based on their titles and abstracts. Three members of the review team (HB, LAN, and JD) screened the titles and abstracts against the inclusion criteria. The remaining full texts were screened by 6 members of the review team (HB, LAN, JD, SL, BM, and LN). At this stage, 20% of the texts were screened by at least 2 reviewers independently to ensure interrater reliability (intraclass correlation coefficient range 0.88-0.96). Any disagreements between the 2 reviewers were resolved through discussion with the wider review team.

### Data Extraction

Data were extracted by 1 reviewer (HB or LAN) and reviewed for accuracy and completeness by another. After verifying all the extracted data, discrepancies were resolved through discussion or adjudication by another party (MF). Extracted data included information on study characteristics (eg, authors, publication year, country, study design, and study population), intervention characteristics (eg, characteristics of the technology, app name, therapeutic modality, and intervention outcomes), and implementation constructs (eg, implementation objectives and implementation results). Textbox 1 provides a description of the implementation outcome criteria.

**Description of implementation variables.**

**Outcome and definition**

- Coproduction: user involvement in the development or evaluation of the intervention through coproduction or another patient and public involvement activities.
- Acceptability: perception among stakeholders that a given evidence-based practice is useful, agreeable, palatable, or satisfactory.
- Appropriateness: appropriateness is the perceived fit, relevance, suitability, or compatibility of an innovation with a practice setting or context.
- Feasibility: actual fit, utility, or suitability and the extent to which an evidence-based practice can be successfully used or conducted within a given context.
- Fidelity: extent to which an evidence-based practice is being delivered as intended. This includes adherence and the quality of program delivery.
- Adoption: intention, decision, or initiation to use or uptake an evidence-based practice.
- Engagement: user enrollment, attendance, session participation, homework completion, adherence, and dropout.
- Penetration: spread, reach, and integration of an evidence-based practice in “real-world” settings.
- Implementation cost: costs associated with implementing an evidence-based practice. This includes cost-effectiveness and cost-benefit.
- Sustainability: uptake by users and the extent to which a newly implemented evidence-based practice is maintained and continued within a service setting’s ongoing, stable operations.

### Quality Assessment

The mixed methods appraisal tool (version 2018) was used to assess the methodological quality of the included studies [33]. It was developed by combining the core relevant methodological criteria found in different well-known and widely used qualitative and quantitative critical appraisal tools. It consists of 2 screening questions applicable to all types of study design and a further 5 questions applicable to specific study designs. Responses were rated on a categorical scale as “no,” “unclear,” or “yes” to any of the methodological quality criteria. Quality assessments were made by 1 of 3 reviewers (SL, BM, and LN). We did not exclude any studies based on quality assessment scores.

### Data Synthesis and Analysis

The extracted data were collated and summarized to produce a narrative summary of the study, sample, and intervention characteristics. To determine the proportion of apps that were sustained or adopted after development, we contacted the corresponding author of the included articles to complete a brief survey about the development and implementation of the app described in their study (Multimedia Appendix 2). If they did not respond, the first or lead author was then contacted. In addition, we searched to check the availability and discoverability of the app in the Apple App Store (iPhone or Mac) and Google Play Store. A codebook approach was used to code and synthesize implementation data from all available sources according to the 10 implementation outcome categories [34].

## Results

The systematic search identified 34 studies published between 2011 and 2021, corresponding to 29 unique apps that reported a beneficial intervention effect when compared with a control group. Figure 1 provides additional details on the screening and inclusion processes.

### Study Characteristics

The characteristics of the included studies are presented in Multimedia Appendix 3 [35-68]. Of the 34 studies, 11 (32%) studies were conducted in the United States; 6 (18%) in the United Kingdom; 3 (9%) in Australia; 2 (6%) each in Italy, Japan, New Zealand, and Spain; and 1 (3%) each in Canada, Iceland, Iran, Israel, South Korea, and Sweden. Most studies (31/34, 91%) were published between 2018 and 2021. Regarding the evaluated sample populations, most studies (24/34, 71%) primarily recruited university students, followed by clinical samples (5/34, 15%), general population samples (5/34, 15%), school students (3/34, 9%), primary care patients (1/34, 3%), and those attending youth organizations (1/34, 3%). In terms of study design, 74% (25/34) of studies were randomized controlled trials (RCTs), 15% (5/34) were pilot RCTs, 6% (2/34) were quasi-experimental, and 6% (2/34) were feasibility trials. Notably, 29% (10/34) of the included studies evaluated the effectiveness of a commercially available app that was not developed by the evaluation study team.

### Intervention Characteristics

Multimedia Appendix 4 [35-68] outlines the format and delivery of interventions assessed in the included studies. Of the 34 studies, 15 (44%) studies aimed to treat mental health problems or reduce symptoms, 14 (41%) aimed to promote well-being or mental health, and 5 (15%) aimed to prevent the onset of mental health problems. Most apps studied (29/34, 85%) were stand-alone, and the remainder (5/34, 15%) accompanied other therapeutic interventions.

### Successful Implementation

The measurement of successful implementation, such as sustained use following the end of the research trial either commercially (eg, discoverable in app stores) or otherwise (including available to young people via schools or mental health services) varied based on unique features of the app itself, its recipients, and its context. To ascertain the proportion of apps that were sustained after development, we contacted the authors of the included articles to request additional information about the implementation of the app reported in their study. We did not contact the authors of the 18% (6/34) of studies testing existing apps that we knew were commercially available and discoverable to the public at the point of the review (eg, Headspace and Calm); that is, being successfully sustained. Of those contacted (28/34, 82%), we collected 23 survey responses (Multimedia Appendix 5 [35-43,48,50,51,54,55,57-61,63,65-67]), and 5 authors did not respond. However, on reviewing the survey responses, 17% (4/23) were evaluating other apps already available. In the absence of survey data for 5 studies, we checked if they were commercially available; 2 (40%) were discoverable on either the Apple App Store (iPhone or Mac) or Google Play Store (Google Inc) [45,62], and 3 (60%) were not available [52,53,64].

In summary, 10 articles evaluated the effectiveness of an existing app that was available for use at the time of the evaluation study [38,44,46,47,49,54,56,59,60,68]. Of the 10 articles, 8 (80%) evaluated an app still available on the market: Headspace [44,46,47,68], Calm [49], Pacifica/Sanvello [38], Smiling Mind [47], Stop Breathe Think [56], and Thrive [59]. The 2 major smartphone app markets (ie, App Store and Google Play) publicly list app ratings out of 5 on their store pages. Google Play also provides download count estimates. The consumer app ratings on a 5-point scale, from the App Store and Google Play, respectively, are as follows: Headspace (rating 4.9 and 4.25) and Calm (rating 4.8 and 4.25) were the most popular (>10 million downloads), followed by Sanvello (rating 4.8 and 4.5), Stop Breathe Think (rating 4.8 and 4.5), Smiling Mind (rating 4.5 and 3.75; >1 million downloads), and Thrive (rating 5.0 and 3.5; >50,000 downloads). Finally, 2 studies evaluated consumer apps that are no longer available: DeStressify [54] and Lantern [60]; therefore, their store statistics are not reported.

Of the 24 studies reporting on a newly developed app, 43% (9/21) are currently available, commercially or otherwise (eg, in mental health services), and 57% (12/21) are no longer available. Most respondents reported that it took several years to develop and test the app reported in their articles, ranging from 6 months to 6 years.

### Markers of Successful Implementation

#### Adoption

Of the 20 apps sustained after development, several are available noncommercially and freely to users in local contexts, including in mental health services [37,42], for university students [36,59], and for corporate organizations [63]. Apps are also accessible to users via commercial channels, including Apple App Store (iPhone or Mac) and Google Play Store (Multimedia Appendix 6 [35-68]).

#### Coproduction

A total of 9 unique apps were reported as being coproduced with young people, 5 of which are either currently available or were previously available following the study but no longer available. The level of youth involvement in the coproduction of the apps varied across studies but involved activities such as a web-based survey, which was delivered to 150 young people (ie, the target end users) [61,69]; market research and beta testing [54]; design workshops with 15 key stakeholders, followed by a series of in-depth interviews [67,70]; focus groups with young people with lived experience that guided the development of app functionalities [42]; and study groups with teenagers and young people who were involved in all developmental phases of the app [43]. Coproduction data were not reported in 12 studies.

#### Acceptability and Appropriateness

Acceptability was generally well assessed with a variety of measures using both qualitative and quantitative methods (*k*=16). Most studies of implemented apps reported them as acceptable, with high user satisfaction and ease of use among young people and health care providers [43,45,61,69]. O’Dea et al [69] examined adolescents’ attitudes toward the concept of a mobile phone app for relationship help and support and reported that, overall, 60.7% (91/150) were likely to use an app for relationship problems, and this was not associated with demographics or social support (*P*>.05). Notably, the likelihood of app use was found to be influenced by the perceived need for help, personal beliefs about app effectiveness, and whether the app was engaging and easy to use. Overall, adolescents found the proposed app content helpful, with an average of 99.3% (149/150) rating the strategies provided as somewhat to very helpful. More than 90% of respondents reported that the app was enjoyable, easy to use and understand, and that they would recommend it to a friend [61,69]. The barriers most commonly experienced were mismatched need, forgetfulness, and being time-poor [61,69]. Acceptability was also assessed for the “Personalized Real-time Intervention for Motivational Enhancement (PRIME)” app designed to improve motivation in young people with early-onset schizophrenia during an exit interview 12 weeks after the trial. Participants rated their satisfaction with specific features of the app, such as the ability to interact with peers and the different goal categories, on a scale from 1 (not at all) to 10 (very much) [67]. Schlosser [67] reported that participants rated their overall satisfaction with “PRIME” highly. Similarly, Broglia et al [38] explored the feasibility and acceptability of supplementing college counselling with the “Pacifica” app and whether this, in turn, had positive clinical outcomes. This blended approach to their intervention was shown to be acceptable and feasible and showed the potential to maintain clinical improvement in anxiety following the completion of a brief counselling intervention [38]. Egilsson et al [43] assessed acceptability with the Systematic Usability Scale, a widely used and relatively well-studied 10-item questionnaire on app usability, where scores range from 0 to 100, and a total score of >70 indicates satisfactory usability and user acceptance [71]. The mean total score on the Systematic Usability Scale was satisfactory (mean 78.09, SD 9.82), indicating adequate usability of the app they tested to improve the emotional and physical health of adolescents [43]. Acceptability data were unavailable for 53% (18/34) of studies, and appropriateness data were unavailable for 68% (23/34) of studies.

#### Feasibility

The extent to which interventions were feasible (ie, the actual fit or practicality) was reported in 35% (12/34) of studies that used a broad range of metrics as indicators of utility and suitability, including log-in frequency, app activity, average number of sessions, recruitment duration, treatment preference, the percentage of participants who completed follow-up assessments, and randomization acceptability [38,42,43,56,67]. To evaluate the feasibility of “PRIME,” the authors examined the log-in frequency, challenges completed, spontaneous and goal achievement moments, peer and coach interactions, and active use rate [67]. Participants in the “Stop, Breathe and Think” trial, which evaluated a publicly available mindfulness app, provided high satisfaction ratings and reported regular use of the app, particularly in the first 2 weeks. However, the rate of recruitment was slow over the course of an academic year, indicating potential feasibility and long-term sustainability concerns [56]. Of the 34 studies, feasibility data were not available for 22 (65%) studies.

#### Fidelity

Fidelity, the extent to which the interventions were delivered as intended, is a less-prominent implementation determinant for apps, given the content control inherent in the structure of the delivery mechanism. However, it is relevant, for example, in one of the included studies, where fidelity outcomes were reported in transcripts from counselling audio recordings where the app was provided as an adjunct to face-to-face counselling [38]. Transcripts were scored to assess the following criteria: (1) number of times the app was discussed, (2) duration of app discussion, (3) whether therapist reviewed client app use, (4) number of app features therapist suggested, and (5) missed opportunities to discuss client app use [38].

#### Engagement

Engagement-related factors, including user enrollment, attendance, session participation, homework completion, adherence, study retention, and dropout, were widely reported outcomes across studies. Engagement data were reported in all but 1 study [37]. A consistent and noteworthy finding across several studies was that engagement decreased over time [43,54,67]. Unsurprisingly, young people identified that accessibility and engagement issues, including user experience, influenced their likelihood of using the intervention [61,69]. For example, engagement with 1 app dropped after the first month of the trial before leveling out over the second and third months [67]. Egilsson et al [43] noted a decrease in average exercises performed between the first week of the intervention and subsequent intervention weeks, with a significant 76% decrease in the total number of in-app health exercises from week 1 to week 2.

A total of 10 articles evaluated a consumer (ie, available on the App Store or Google Play) smartphone app at the time of their publication. Data collection methods and the degree of detail for app use differed across studies. Most studies (6/10, 60%) collected information regarding app use from participants’ self-reports [38,44,47,54,59,68], followed by passive activity tracking (2/10, 20%) provided by the official app teams [46,49], combined self-report and passive tracking (1/10, 10% [56]), or not specified (1/10, 10% [60]). Self-report measures were generally 1-item data points, varying from 10-point scales between “did not use at all” and “used as often as requested” [54] to number of times per week [56,59] and yes or no daily measures [47]. In total, 2 studies requested an in-app summary screenshot of completed minutes as a record [44,68], and Yang et al [68] further provided a paper calendar for record tracking. On the other hand, passive activity tracking provided greater detail, including the date, time, and duration of an in-app exercise [46,49,56]. Newman et al [60] did not specify app use data collection methods but recorded more details of use than other studies, such as total visits on the app, number of sessions on the app, and minutes on the app. It is noteworthy that Yang et al [68] reported that individuals in the experimental group who tried Headspace at least once had an average of 12 days of use over the course of their 30-day, laissez-faire (ie, use this app as you would normally) intervention without additional prompts or ongoing accountability. In addition, 74% of those who used the app during the 30-day intervention period continued to use it for an additional 30 days. These findings imply that motivating individuals to use an app just once may be an important step in retention.

Although app use measures provide straightforward indicators of attendance, session participation, homework completion, and adherence are unclear owing to variations in their definitions. Session participation may be synonymous with attendance for some researchers, whereas others operationalize it as meaningful, back-and-forth interactions between a user and agent (eg, app, coach, or counselor). A total of 20% (2/10) of studies reporting on consumer apps included an agent (therapists [38] and coaches [60]) as part of their intervention, but only 1 analyzed whether user-agent communication (ie, session participation) affected efficacy. Newman et al [60] reported “treatment usage” (a composite variable including user-agent interaction), which did not affect symptom outcomes. In addition, homework completion may be defined as completing the prescribed exercises in a study, whereas another study may define it as tasks in addition to the primary intervention. None of the included studies mentioned “session participation” or “homework.” In terms of adherence, 50% (5/10) of studies explicitly measured adherence (eg, app use data, including time spent and sessions using the app) [46,47,49,54,59]. An additional study [56] did not operationalize “adherence,” although they collected characteristic adherence data through self-report, asking users how many days in the past 2 weeks they had used the app. However, given the lack of conceptual clarity and consensus on adherence, it is difficult to assess whether adherence affects clinical health outcomes in the context of consumer app evaluation.

#### Implementation Cost

Data on the costs associated with implementing the apps, including cost-effectiveness and cost-benefit, are not readily available in the public domain. However, results from the survey indicate that, unsurprisingly, apps developed from scratch were the costliest to develop and test. Notably, several apps included in this review were developed from existing, ready-made platforms, which were modified for different interventions rather than building the app from the ground up. For example, the GGtude platform was developed in 2016 and hosts several apps designed for different populations and presenting problems [35,40,41,66]. This approach has been successful with several studies reporting that daily use of apps from the GGtude platform during a period of 2 weeks (3 minutes a day) is associated with significant beneficial effects on mental health in nonclinical and subclinical samples [40,41,66]. Similarly, Levin et al [55] have focused their efforts on developing app prototypes rapidly using easy-to-use website platforms to customize intervention content and develop generalizable knowledge about principles and processes that work in mobile apps rather than building and developing new apps from scratch [55,57]. However, the authors note that their platform was not set up for commercialization or broad deployment because the app is delivered within the LifeData system and lacks key features needed for a public launch, including budget and support for ongoing technical maintenance and monitoring user inquiries and data. It was also noted that ongoing updates are likely needed to remain relevant and competitive with other market products, which involves undertaking regular market scans and content refinement to ensure the product remains well positioned and effective in an increasingly saturated market [61].

Another approach taken by the authors of the included studies was to evaluate existing, publicly available apps for a specific local context and also as a potential adjunct to existing in-person therapies (eg, university students and college counselling centers) [38,54,56,59,60]. For example, Broglia et al [38] contacted the developers of a mood monitoring app to test its use in conjunction with usual care counselling sessions. Similarly, Levin et al [56] conducted a pilot RCT to evaluate the feasibility and acceptability of a publicly available mindfulness app for university students. Using existing, commercially available apps or building prototypes using adaptable web platforms provides a lower cost and quicker alternative to developing and evaluating new apps from the ground up. However, implementing apps in distinct local contexts requires well-thought-out and tailored implementation strategies, with consideration given to common barriers, especially acceptability and feasibility.

#### Sustainability and Penetration

Sustainability can be measured in several ways, including apps being fully integrated into service settings and a steady budget for app advertising, maintenance, and updates [21]. The number of app downloads and interactions over time also provides an indication of sustained uptake over time. However, none of the included studies reported data on the sustainability of the interventions evaluated in their study. As such, the literature is sparse regarding the long-term integration and penetration of mobile interventions within mental health and other support service settings.

### Study Quality

Methodological quality varied across the included studies (Multimedia Appendix 7 [35-68]). Most studies (32/34, 94%) were judged as having possible limitations in at least 1 criterion. Most studies (29/34, 85%) clearly described the randomization of the study participants or the process for recruiting a representative sample. Most randomized trials reported complete outcome data (30/32, 94%) and described samples that were comparable at baseline (27/32, 84%). However, few studies described the process used to blind the outcome assessor to the intervention group (10/32, 31%). Only half of the studies (16/32, 50%) reported acceptable adherence rates to the intervention. Regarding the 2 nonrandomized trials, both studies used appropriate outcome measures. Of the 2 studies, 1 (50%) was judged as not clearly accounting for confounders in the study design and analysis. Both studies were judged as adhering to the intervention protocol [38,53].

## Discussion

### Principal Findings

The primary aim of this systematic review was to determine the proportion of evidence-based mental health and well-being apps that have been successfully adopted and sustained in “real-world” settings. In total, 29% (10/34) of studies were identified that evaluated the effectiveness of 8 existing, commercially available mental health apps. The remaining 71% (24/34) of included studies evaluated 21 newly developed apps, of which 43% (9/21) are currently available, commercially or otherwise (eg, in mental health services), and 57% (12/21) were no longer available at the time of enquiry. Therefore, these results not only indicate a 43% implementation success rate of new apps but also provide some information on how existing efficacious commercial apps are promoted and sustained in the field.

Broadly synthesized using 10 dimensions of implementation, our review suggests that measures of adoption, acceptability, appropriateness, and feasibility are more frequently reported than indicators of cost, fidelity, sustainability, and penetration. Implementation outcomes were unavailable for many of the studies, precluding direct comparisons between those apps that were implemented and those that were not across constructs. To partially address our second research question, most of the apps that had been implemented confirmed a degree of adoption, acceptability, appropriateness, feasibility, and engagement. These determinants are relevant to a range of interventions and would benefit from broad systematic incorporation into the development of a smartphone app. Other important factors identified included the coproduction of interventions with young people and the need to embed apps within local settings, such as schools, universities, and mental health services, rather than relying on commercial strategies. Although assessing implementation outcomes using an integrated framework allows for a more systematic assessment of outcomes, the review highlights a lack of measurement precision around implementation constructs in that an array of overlapping terms, such as acceptability and usability, are often used interchangeably, highlighting the need for greater consensus on how to measure and report implementation determinants. There is also a need to elucidate the relationship between different implementation constructs (eg, acceptability and engagement) and intervention effectiveness (eg, clinical change) [22]. Modeling these relationships was not possible in the current review because of data quality issues. Further empirical research is needed to model the interrelationships between implementation variables to better understand the nature of their connections and their impact on implementation success.

Confirming acceptability and engagement in the early phases of intervention development, before implementation commences, is an important step in ensuring that the intervention has potential longevity and may be important for saving time and money. As evidence suggests that engagement is the highest in the first days or weeks of downloading an app, it might be more appropriate for apps to be developed with this anticipated behavior in mind [72].

Research has identified that intervention- and person-specific factors that influence engagement with mobile mental health interventions should be considered [25]. For example, getting individuals to use an app just once is an important step in retention. It is likely that an initial hook is needed to catch the attention of young people to encourage them to identify and download the app. Thereafter, rewards and gamification are reported by young people as motivating factors for ongoing engagement and retention [25]. Similarly, usability has been reported as important for promoting engagement. Acceptability and engagement are critical elements of mental health apps that support users [73]. However, it is recognized that operationalizing meaningful app engagement is not straightforward, as many downloaded apps are never used and more work is needed to define sustained engagement, what leads to it, and how to create products that achieve it [73,74]. Further research may explore characteristics facilitating initial use, continued use, and the implications of changing the treatment structure during an intervention (eg, Do individuals find it motivating or overwhelming?).

### Barriers to Successful Implementation

The importance of identifying mental health interventions that are efficacious needs to progress in concert with addressing implementation requirements from the outset of app development. This can be a complicated focus for researchers and funding bodies, as in the absence of efficacy, it might seem premature to address dissemination issues, but given the high redundancy of scientific studies—less than half of the efficacious apps were actually identified as being in sustained use—these questions must be paramount at the outset of the research cycle.

In addition to the markers of successful implementation, there are several extant barriers, including high cost, time, funding constraints, and lengthy research processes. The process of applying for funding, app development, data collection, data analysis, and app release is lengthy and expensive, taking up to 6 years [39]. The sustainability and penetration of apps is contingent on projects being funded or grants being successful, and even when funding is secured, it is often time limited [42,67,75]. The way in which funding streams are set up means that it is harder to secure funding for the follow-on implementation research than for the initial evaluation project.

Other challenges include the continuous, rapidly evolving development of technology, which results in the need for continued code updates and upgrades to allow for compatibility with smartphone operating systems [42,61]. Additional noted barriers include the rapidly changing nature of many commercial providers supplying the app technology, sometimes with significant staff movement, changes to the focus of work depending on commercial drivers, and short life spans of some tech companies [60]. Given that only a small proportion of existing, commercially based apps are being well sustained, rather than developing de novo apps, it might be that evaluating more successful apps and adapting them to specific contexts can propel implementation in the field. However, it is recognized that the commercial app marketplace is highly competitive, and research has identified that, based on our estimates of monthly active users, mindfulness and meditation apps appear to be the most popular: Headspace and Calm account for 13.4 million users, Replika and Wysa account for 1.5 million users, and Reflectly and Daylio account for 840,000 users [76]. In total, these 6 apps monopolize the marketplace, many of which have been made freely available to young people and extensively marketed; hence, they penetrate the market and account for 83% of the monthly active users of mental health and wellness apps [76]. As noted by others, currently, the mental health and well-being apps that have been rigorously evaluated struggle to attract users, and apps with many users are rarely evaluated [76,77].

There are several possible explanations for the success of these commercial apps. Fish and Saul [44] reported that Headspace, one of the most popular consumer apps in the market, applies gamification techniques, such as accomplishment, empowerment, social influence, and ownership to improve engagement and motivate individuals to enjoy meditations and find them rewarding. Several engagement design features in Headspace, such as user tracking, reminder function, and push notifications, can improve engagement, these features were not actively part of any of the evaluated studies and were left for use at the discretion of the individuals [46]. However, Huberty et al [49] report that these features may be critical for engagement and acceptability.

### Groups at Risk of Exclusion

An interesting finding of this review is that there are certain groups that are less likely to access mental health and well-being apps. It is noteworthy that most studies included in this review recruited university students (24/34, 71%). There was a marked absence of youth samples from underserved or marginalized populations, including but not limited to migrants, asylum seekers and refugees, those experiencing homelessness, and those from socioeconomically deprived backgrounds. These potentially high-risk groups are typically underrepresented in research, face access and engagement barriers when navigating health care systems, and experience digital exclusion [78]. The studies included in this systematic review had not targeted these populations, and research exploring the acceptability, appropriateness, and feasibility of app-based interventions is lacking.

### Limitations

Although this review was rigorous, carefully executed, and used a robust methodological approach, it was not without limitations. Foremost, although the review team attempted to identify and include as many articles as possible, some articles may have been missed because of the inconsistencies in how implementation outcomes are recorded and reported. It was also difficult to ensure that all apps for this age group were identified because those aged between 15 and 25 years are harder to differentiate in adolescent and adult studies, meaning we might have missed some relevant studies. Although the focus of this review was young people aged between 15 and 25 years, most of the included studies recruited university students, and most of the study samples had a mean age of >20 years. This finding suggests that there may be a lack of effective mental health apps targeted at mid to late teenagers. This may be a result of the ethical constraints of recruiting young people aged <18 years (16 years in the United Kingdom) to research studies, as it is easier to recruit those who are deemed able to independently consent to trial participation. Additional research is needed to determine whether mental health apps have been developed for this group; however, upon evaluation, they were deemed ineffective. Given the considerable heterogeneity in the social, emotional, and cognitive development and maturity of young people in this age range, research is needed to understand the potential differential impacts and utility of apps throughout adolescence and young adulthood.

In addition, the review included several apps that were delivered as an adjunct to in-person therapies. It is recognized that the reported successful implementation of these apps may in fact be a by-product of the success of the primary therapeutic intervention rather than the app itself. Research comparing face-to-face therapies with and without additional app support provides evidence to support the superiority of adjunctive interventions compared with standard intervention-only conditions [79]. However, additional research is needed to enable us to determine the role of apps in the successful implementation and sustainability of blended intervention programs.

Unpublished data were not included in the search, which may have affected the results of this review. Nevertheless, this approach was also seen as a further strength by ensuring that only peer-reviewed interventions were included. Given that the focus of our review was on apps that had been found to be efficacious, the publication bias effect was minimal. However, it is possible that companies that have developed the apps possess valuable data pertaining to some aspects of implementation. Commercial companies are likely to have collected a wealth of relevant and informative data on user engagement and app use over several years. However, owing to competing commercial interests and a lack of regulation in the mobile mental health app arena, these data are not publicly available or made openly accessible to researchers [80]. Nonetheless, an attempt was made to obtain implementation data when it was not included in the articles by contacting the study authors, most of whom responded.

Finally, more than two-thirds of the apps were not independently evaluated and, therefore, an important consideration is that “developer bias” may have impacted the analysis and subsequent findings of those trials. More independent and robust evaluation of apps is needed, and findings must be shared in a manner that is accessible to the scientific community as well as the users of the app. This will require time and resources but needs to become integral to the development process to mitigate potential bias in evaluations moving forward [81], be that within purely commercial or research contexts or for commercial-research collaborations.

### Recommendations for Research and Practice

#### Coproduction With Young People

Coproducing interventions with young people may help improve the acceptability and feasibility of the end product, which, in turn, can improve intervention effectiveness [25,26]. Coproduction actively involves relevant stakeholder groups in the design process to help ensure that the technology developed meets their needs and is usable. In coproduction, intended users work with designers, developers, and researchers during the innovation and development process [82]. A range of methods have been used to enact young people’s involvement in health research, often under the umbrella of “Young People’s Advisory Groups” (YPAGs) [83]. This includes, but is not limited to, market research and beta testing, design workshops, in-depth interviews, and focus groups. Consistent reporting on the methods of involvement and outputs of YPAGs in publications will help develop a better understanding of the influence of YPAGs in youth mental health research, enabling better systems for meaningful youth involvement in research [83,84].

#### Cost-effective Prototyping

To address the lengthy and costly research and evaluation processes, adaptable and modifiable interventions can be developed from existing platforms, which can then be delivered quickly in a personalized way to meet a range of users’ needs. As noted by others, this is complicated by the need to balance scientific rigor with the fast pace with which technology advances to achieve the adoption of evidence-based practice [85]. To address some of the time, funding, and financial burdens, it may be that researchers and clinicians work collaboratively with industry partners to capitalize on existing interventions and platforms, modifying and evaluating them for their local context. However, it is acknowledged that working with developers involves its own set of complex challenges. Often, researchers invest time and resources in the initial stages in building prototypes with developers only to find out that the intervention is ineffective, not feasible, or not economically viable. As an alternative, researchers can take advantage of free or low-cost systems for rapid prototyping, such as Qualtrics, or an Ecological Momentary Assessment platform, such as LifeData or MetricWire.

#### Assess and Report Implementation Outcomes

Intervention evaluation should include key implementation determinants, such as acceptability, feasibility, appropriateness, and cost. Currently, implementation outcomes are poorly reported, and publications reporting the results of RCTs focus on clinical outcomes, often neglecting sustainability, cost, and the process of embedding the intervention into “real-world” clinical practice. It has been reported that psychological intervention articles report, at most, 64% of the information needed to implement interventions [86]. It is important that researchers use consistent implementation measurement and reporting to allow meaningful and accurate comparisons across studies and share the information required by implementers. Assessing implementation outcomes using a conceptually grounded framework allows for a more systematic assessment of outcomes while supporting the rigor and reproducibility of implementation research and providing the building blocks for implementation evaluation.

#### Hybrid Intervention Models

There has been increased attention toward “blended” approaches, where web-based support is provided as an adjunct to, rather than a replacement for, face-to-face treatment. The importance of human support in web-based therapies and the perceived value of blending mobile health interventions with traditional face-to-face treatment is well described in the literature [87-89]. A blended approach has the potential to reduce the relative load of costly face-to-face contact while boosting engagement, enhancing outcomes, and increasing treatment acceptability [88].

#### Early Consideration of Economic Sustainability

To support the implementation of evidence-based and efficacious interventions, early consideration of funding and costs are crucial. Funding bodies should endeavor to develop new, responsive funding streams, including implementation-specific grants, to focus on the implementation of existing evidence-based interventions, as the translation of evidence from feasibility to adoption is poorly realized, bringing considerable redundancy to the field of intervention research. Currently, it is harder for researchers to secure funding for follow-on work than for the initial evaluation project, and it is not possible to secure funding ahead of time for this in the initial app, as they are usually feasibility studies, and so effectiveness cannot be assumed. However, services are unable to adopt evidence-based practices until implementation drivers are tested and addressed. Furthermore, it is important that a clear business model is planned and in place, with consideration given to the potential market and how the implementation of the product will take place and any anticipated revenue generation [85].

#### Involvement of Vulnerable Groups

As in other areas of mental health research, young people from marginalized and underserved groups (eg, from low-income backgrounds, refugees, or asylum seekers; those not in education, employment, or training; members of ethnic and sexual minorities; and those under state care) were underrepresented in these studies, which typically focused on university students. Few attempts have been made in the literature to create new interventions or to adapt existing ones to meet the complex and heterogeneous needs of these young people [90]. Research exploring the acceptability, appropriateness, and feasibility of mental health app–based interventions for this group is lacking [89]. Work to assess the acceptability and feasibility of mental health apps for underserved young people is needed to ensure that they are not further excluded from research and to advance toward mental health provision that meets their support needs.

### Conclusions

Despite the significant amount of funding that has been directed toward the development of mobile mental health interventions, few have published evidence-based data to support their use in real-world settings, and even fewer have been successfully transitioned into sustainable mental health interventions. Although it had been thought that smartphone apps held the potential to address many of the current issues facing service provision in youth mental health by improving the scalability and affordability of evidence-based mental health interventions for young people and addressing health disparities by providing wider access to underserved populations, more work is needed to improve key implementation drivers, such as uptake and adoption. Innovative and targeted funding mechanisms that are quick, responsive, and encouraging of broad stakeholder and industry partnerships, where data are openly shared, are essential to ensure mental health and well-being app development, evaluation, implementation, and sustainability proceeds in a direction that will enable evidence-based interventions to be made available quickly to young people who may benefit from them.

## Appendix

Multimedia Appendix 1 Systematic search strategy.

Multimedia Appendix 2 Author survey questions.

Multimedia Appendix 3 Study characteristics.

Multimedia Appendix 4 Intervention characteristics.

Multimedia Appendix 5 Implementation and sustainability.

Multimedia Appendix 6 Implementation variables.

Multimedia Appendix 7 Mixed methods appraisal tool.

## Abbreviations

PRIME: Personalized Real-time Intervention for Motivational Enhancement
PRISMA: Preferred Reporting Items for Systematic Reviews and Meta-Analyses
RCT: randomized controlled trial
YPAG: Young People’s Advisory Groups

## Data Availability

The data extracted to support the findings of this review can be obtained from the corresponding author on reasonable request.
