# Supplementary material for: Determination of Markers of Successful Implementation of Mental Health Apps for Young People: Systematic Review
Source: J Med Internet Res. 2022 Nov 9;24(11):e40347. doi: 10.2196/40347 (PMC9685513; doi:10.2196/40347)
Supplement: Multimedia Appendix 1 [file jmir_v24i11e40347_app1.docx]

Multimedia Appendix 1: Systematic search strategy

Search strategies run on 02/02/2021 by Eli Harriss, a librarian at the Bodleian Health Care Libraries, University of Oxford. Please acknowledge my support in publications related to these searches as a co-author.

## Search Results

| Ovid Embase | 9100 |
| --- | --- |
| Ovid Medline | 6282 |
| Ovid PsycINFO | 3317 |
| Cochrane CENTRAL | 6393 |
| Cochrane Database | 1219 |
| Total | 26311 |
| Total after deduplication | 18577 |

## Search Strategies

**Database: Embase 1974 to present**

Search Strategy:

--------------------------------------------------------------------------------

1 (minor* or boy* or girl* or schoolchild* or pupil* or adolescen* or juvenil* or youth* or teen* or pubescen* or prepub* or pre-pub* or preadolescen* or pre-adolescen*).ti,ab. (1248331)

2 ("young adult*" or "young people" or "young person" or "young man" or "young men" or "young woman" or "young women" or student*).ti,ab. (601011)

3 juvenile/ or exp adolescent/ (1591279)

4 young adult/ (389885)

5 1 or 2 or 3 or 4 (3017871)

6 exp mental health/ (168731)

7 child psychiatry/ (20956)

8 exp mental disease/ (2258212)

9 exp community mental health service/ (326)

10 exp intellectual impairment/ (526206)

11 mental patient/ (26819)

12 exp "psychological and psychiatric procedures"/ (1269795)

13 ("mental difficult*" or "mental health challenge*" or "mental health difficult*" or "mental health problem*" or "mental challenge*" or "mental well$being" or "mental health well$being" or "mental illness" or "mental health illness" or "mood disorder*").ti,ab. (82954)

14 ("mental health" or "attention deficit disorder" or ADHD or "behavio$r disorder*" or "communication disorder*" or "conduct disorder*" or "emotional adjustment*" or "emotional* disturb*" or "intellectual development disorder*" or "learning disorder*" or "psychiatric patient*" or psychopatholog* or suicid* or "thought disturbance*" or psychotherap* or "child psychopatholog*").ti,ab. (444441)

15 (psychiatr* or anxiety or depression or suicid* or stress or psychos* or psychotic* or bipolar* or schizophren* or schizoaff* or "eating disorder*" or bulemi* or anorexi*).ti,ab. (2158482)

16 6 or 7 or 8 or 9 or 10 or 11 or 12 or 13 or 14 or 15 (4236006)

17 exp mobile phone/ (32031)

18 exp microcomputer/ (14721)

19 ((handheld or "hand held") adj1 (computer* or pc*)).ti,ab. (839)

20 ("cell* phone*" or "mobile phone*" or smartphone* or "smart phone*" or "personal digital assistant*" or PDA).ti,ab. (50478)

21 personal digital assistant/ (1555)

22 "palmtop computer*".ti,ab. (100)

23 (tablet adj3 (device* or comput*)).ti,ab. (2322)

24 (blackberry or nokia or symbian or (windows adj3 (mobile* or phone*)) or INQ or HTC or sidekick or android or iphone* or ipad* or samsung).ti,ab. (15145)

25 social media/ (24459)

26 exp internet/ or exp mobile application/ (127545)

27 ((internet* or app or apps or application* or "mobile device*" or "mobile app*" or smartphone* or "mobile phone*" or digital* or technolog* or computer* or mhealth* or mhealth or mobile health or ehealth or "electronic health" or "social media" or phone or online or web-based) adj4 (treatment* or intervention* or therap* or training or support* or help* or assistan*)).ti,ab. (184751)

28 17 or 18 or 19 or 20 or 21 or 22 or 23 or 24 or 25 or 26 or 27 (383742)

29 5 and 16 and 28 (17341)

30 "phase 3 clinical trial (topic)"/ or exp clinical trial/ or "randomized controlled trial (topic)"/ (1773844)

31 (development or evaluation or trial* or RCT* or evidence or efficacy or effectiveness or implementation or case-control or fidelity or assessment or "clinical stud*" or testing or acceptability or feasibility or adoption).ti,ab. (10047498)

32 intervention*.ti,ab. (1465682)

33 30 or 31 or 32 (11232368)

34 29 and 33 (10928)

35 34 (10928)

36 limit 35 to yr="2011 -Current" (9100)

**Database: Medline (Ovid MEDLINE® Epub Ahead of Print, In-Process & Other Non-Indexed Citations, Ovid MEDLINE® Daily and Ovid MEDLINE®) 1946 to present**

Search Strategy:

--------------------------------------------------------------------------------

1 (minor* or boy* or girl* or schoolchild* or pupil* or adolescen* or juvenil* or youth* or teen* or pubescen* or prepub* or pre-pub* or preadolescen* or pre-adolescen*).ti,ab. (971261)

2 ("young adult*" or "young people" or "young person" or "young man" or "young men" or "young woman" or "young women" or student*).ti,ab. (458036)

3 adolescent/ or young adult/ (2476058)

4 1 or 2 or 3 (3342851)

5 Mental Health/ (41359)

6 adolescent psychiatry/ or child psychiatry/ (6860)

7 Community Mental Health Services/ (18660)

8 exp Community Psychiatry/ (2044)

9 exp Mental Disorders/ (1266972)

10 exp Emotional Adjustment/ (1414)

11 exp Mental Health Services/ (97581)

12 Affective Symptoms/ (13158)

13 exp Intellectual Disability/ (97184)

14 Mentally Ill Persons/ (6229)

15 Psychopathology/ (7451)

16 exp Self-Injurious Behavior/ (72514)

17 exp Psychotherapy/ (199342)

18 ("mental difficult*" or "mental health challenge*" or "mental health difficult*" or "mental health problem*" or "mental challenge*" or "mental well$being" or "mental health well$being" or "mental illness" or "mental health illness" or "mood disorder*").ti,ab. (62023)

19 ("mental health" or "attention deficit disorder" or ADHD or "behavio$r disorder*" or "communication disorder*" or "conduct disorder*" or "emotional adjustment*" or "emotional* disturb*" or "intellectual development disorder*" or "learning disorder*" or "psychiatric patient*" or psychopatholog* or suicid* or "thought disturbance*" or psychotherap* or "child psychopatholog*").ti,ab. (346961)

20 (psychiatr* or anxiety or depression or suicid* or stress or psychos* or psychotic* or bipolar* or schizophren* or schizoaff* or "eating disorder*" or bulemi* or anorexi*).ti,ab. (1676477)

21 5 or 6 or 7 or 8 or 9 or 10 or 11 or 12 or 13 or 14 or 15 or 16 or 17 or 18 or 19 or 20 (2731809)

22 exp Cell Phone/ (11254)

23 exp Computers, Handheld/ (8822)

24 ((handheld or "hand held") adj1 (computer* or pc*)).ti,ab. (696)

25 ("cell* phone*" or "mobile phone*" or smartphone* or "smart phone*" or "personal digital assistant*" or PDA).ti,ab. (37628)

26 Mobile Applications/ (6914)

27 "palmtop computer*".ti,ab. (92)

28 (tablet adj3 (device* or comput*)).ti,ab. (1503)

29 (blackberry or nokia or symbian or (windows adj3 (mobile* or phone*)) or INQ or HTC or sidekick or android or iphone* or ipad* or samsung).ti,ab. (9044)

30 exp Internet/ or Social Media/ (82831)

31 ((internet* or app or apps or application* or "mobile device*" or "mobile app*" or smartphone* or "mobile phone*" or digital* or technolog* or computer* or mhealth* or mobile health or ehealth or "electronic health" or "social media" or phone or online or web-based) adj4 (treatment* or intervention* or therap* or training or support* or help* or assistan*)).ti,ab. (141105)

32 22 or 23 or 24 or 25 or 26 or 27 or 28 or 29 or 30 or 31 (261489)

33 exp Clinical Trials as Topic/ (351838)

34 (development or evaluation or trial* or RCT* or evidence or efficacy or effectiveness or implementation or case-control or fidelity or assessment or "clinical stud*" or testing or acceptability or feasibility or adoption).ti,ab. (7674055)

35 intervention*.ti,ab. (1054790)

36 33 or 34 or 35 (8239059)

37 4 and 21 and 32 and 36 (7576)

38 37 (7576)

39 limit 38 to yr="2011 -Current" (6282)

**Database: PsycINFO 1806 to present**

Search Strategy:

--------------------------------------------------------------------------------

1 (minor* or boy* or girl* or schoolchild* or pupil* or adolescen* or juvenil* or youth* or teen* or pubescen* or prepub* or pre-pub* or preadolescen* or pre-adolescen*).ti,ab. (495279)

2 ("young adult*" or "young people" or "young person" or "young man" or "young men" or "young woman" or "young women" or student*).ti,ab. (596775)

3 exp students/ (269290)

4 1 or 2 or 3 (1043276)

5 exp mental health/ (69585)

6 child psychiatry/ (6836)

7 adolescent psychiatry/ (5221)

8 adolescent psychotherapy/ (2960)

9 exp mental disorders/ (876852)

10 psychiatric patients/ (28769)

11 community psychiatry/ (877)

12 exp emotional adjustment/ (21967)

13 exp mental health services/ (42556)

14 exp well being/ (46269)

15 emotional disturbances/ (9907)

16 exp self-destructive behavior/ (42460)

17 exp thought disturbances/ (21190)

18 exp behavior disorders/ (58172)

19 exp psychopathology/ (37670)

20 psychotherapeutic outcomes/ (5041)

21 preventive mental health services/ (2433)

22 mental health program evaluation/ (2155)

23 abnormal psychology/ (1352)

24 exp mental health programs/ (10927)

25 exp communication disorders/ (60045)

26 conduct disorder/ (4440)

27 ("mental difficult*" or "mental health challenge*" or "mental health difficult*" or "mental health problem*" or "mental challenge*" or "mental well$being" or "mental health well$being" or "mental illness" or "mental health illness" or "mood disorder*").ti,ab. (70647)

28 ("mental health" or "attention deficit disorder" or ADHD or "behavio$r disorder*" or "communication disorder*" or "conduct disorder*" or "emotional adjustment*" or "emotional* disturb*" or "intellectual development disorder*" or "learning disorder*" or "psychiatric patient*" or psychopatholog* or suicid* or "thought disturbance*" or psychotherap* or "child psychopatholog*").ti,ab. (445617)

29 (psychiatr* or anxiety or depression or suicid* or stress or psychos* or psychotic* or bipolar* or schizophren* or schizoaff* or "eating disorder*" or bulemi* or anorexi*).ti,ab. (957525)

30 5 or 6 or 7 or 8 or 9 or 10 or 11 or 12 or 13 or 14 or 15 or 16 or 17 or 18 or 19 or 20 or 21 or 22 or 23 or 24 or 25 or 26 or 27 or 28 or 29 (1631409)

31 exp mobile devices/ (8492)

32 computer applications/ or mobile applications/ (12436)

33 exp social media/ (15633)

34 exp internet usage/ (12927)

35 ((handheld or "hand held") adj1 (computer* or pc*)).ti,ab. (396)

36 ("cell* phone*" or "mobile phone*" or smartphone* or "smart phone*" or "personal digital assistant*" or PDA).ti,ab. (10552)

37 (tablet adj3 (device* or comput*)).ti,ab. (642)

38 (blackberry or nokia or symbian or (windows adj3 (mobile* or phone*)) or INQ or HTC or sidekick or android or iphone* or ipad* or samsung).ti,ab. (2198)

39 ((internet* or app or apps or application* or "mobile device*" or "mobile app*" or smartphone* or "mobile phone*" or digital* or technolog* or computer* or mhealth* or mhealth or mobile health or ehealth or "electronic health" or "social media" or phone or online or web-based) adj4 (treatment* or intervention* or therap* or training or support* or help* or assistan*)).ti,ab. (45136)

40 31 or 32 or 33 or 34 or 35 or 36 or 37 or 38 or 39 (85517)

41 exp clinical trials/ or exp treatment effectiveness evaluation/ (37158)

42 (development or evaluation or trial* or RCT* or evidence or efficacy or effectiveness or implementation or case-control or fidelity or assessment or "clinical stud*" or testing or acceptability or feasibility or adoption).ti,ab. (1771921)

43 intervention*.ti,ab. (394896)

44 41 or 42 or 43 (1935625)

45 4 and 30 and 40 and 44 (4430)

46 45 (4430)

47 limit 46 to yr="2011 -Current" (3317)

**Cochrane Database of Systematic Reviews**

**Issue 2 of 12, February 2021**

**Cochrane Central Register of Controlled Trials**

**Issue 2 of 12, February 2021**

#1 minor* or boy* or girl* or schoolchild* or pupil* or adolescen* or juvenil* or youth* or teen* or pubescen* or prepub* or pre-pub* or preadolescen* or pre-adolescen* 183568

#2 "young adult*" or "young people" or "young person" or "young man" or "young men" or "young woman" or "young women" or student* 119871

#3 MeSH descriptor: [Adolescent] explode all trees 104274

#4 MeSH descriptor: [Young Adult] explode all trees 65387

#5 #1 or #2 or #3 or #4 256906

#6 MeSH descriptor: [Mental Health] explode all trees 1534

#7 MeSH descriptor: [Child Psychiatry] explode all trees 12

#8 MeSH descriptor: [Adolescent Psychiatry] explode all trees 21

#9 MeSH descriptor: [Community Mental Health Services] explode all trees 732

#10 MeSH descriptor: [Community Psychiatry] explode all trees 19

#11 MeSH descriptor: [Mental Disorders] explode all trees 73817

#12 MeSH descriptor: [Emotional Adjustment] explode all trees 60

#13 MeSH descriptor: [Mental Health Services] explode all trees 6852

#14 MeSH descriptor: [Affective Symptoms] explode all trees 453

#15 MeSH descriptor: [Adolescent Health] explode all trees 31

#16 MeSH descriptor: [Communication Disorders] explode all trees 1768

#17 MeSH descriptor: [Intellectual Disability] explode all trees 1429

#18 MeSH descriptor: [Mentally Ill Persons] explode all trees 52

#19 MeSH descriptor: [Psychopathology] explode all trees 84

#20 MeSH descriptor: [Self-Injurious Behavior] explode all trees 1457

#21 MeSH descriptor: [Psychotherapy] explode all trees 24148

#22 #6 or #7 or #8 or #9 or #10 or #11 or #12 or #13 or #14 or #15 or #16 or #17 or #18 or #19 or #20 or #21 91953

#23 "mental difficult*" or "mental health challenge*" or "mental health difficult*" or "mental health problem*" or "mental challenge*" or "mental well$being" or "mental health well$being" or "mental illness" or "mental health illness" or "mood disorder*" 6838

#24 "mental health" or "attention deficit disorder" or ADHD or "behavio$r disorder*" or "communication disorder*" or "conduct disorder*" or "emotional adjustment*" or "emotional* disturb*" or "intellectual development disorder*" or "learning disorder*" or "psychiatric patient*" or psychopatholog* or suicid* or "thought disturbance*" or psychotherap* or "child psychopatholog*" 53899

#25 psychiatr* or anxiety or depression or suicid* or stress or psychos* or psychotic* or bipolar* or schizophren* or schizoaff* or "eating disorder*" or bulemi* or anorexi* 217706

#26 #22 or #23 or #24 or #25 265668

#27 MeSH descriptor: [Cell Phone] explode all trees 1710

#28 MeSH descriptor: [Computers, Handheld] explode all trees 696

#29 MeSH descriptor: [Mobile Applications] explode all trees 686

#30 MeSH descriptor: [Social Media] explode all trees 162

#31 MeSH descriptor: [Internet] explode all trees 4069

#32 ((handheld or "hand held") near/1 (computer* or pc*)) 409

#33 "cell* phone*" or "mobile phone*" or smartphone* or "smart phone*" or "personal digital assistant*" or PDA 8612

#34 "palmtop computer*" 13

#35 tablet near/3 (device* or comput*) 919

#36 blackberry or nokia or symbian or (windows near/3 (mobile* or phone*)) or INQ or HTC or sidekick or android or iphone* or ipad* or samsung 2762

#37 ((internet* or app or apps or application* or "mobile device*" or "mobile app*" or smartphone* or "mobile phone*" or digital* or technolog* or computer* or mhealth* or mobile health or ehealth or "electronic health" or "social media" or phone or online or web-based) near/4 (treatment* or intervention* or therap* or training or support* or help* or assistan*)) 72123

#38 #27 or #28 or #29 or #30 or #31 or #32 or #33 or #34 or #35 or #36 or #37 80494

#39 #5 and #26 and #38 9700

Limited to publication dates 01/01/2011-31/12/2021
