# Supplementary material for: Determination of Markers of Successful Implementation of Mental Health Apps for Young People: Systematic Review
Source: J Med Internet Res. 2022 Nov 9;24(11):e40347. doi: 10.2196/40347 (PMC9685513; doi:10.2196/40347)
Supplement: Multimedia Appendix 2 [file jmir_v24i11e40347_app2.docx]

Multimedia Appendix 2 Author survey questions

**Markers of successful implementation and sustainment of evidence-based mental health apps for young people: a systematic review [CRD42021224365]**

Thank you very much for answering these questions on the implementation of the smartphone app discussed in your study. This should take less than 4 minutes to complete.

1. Please select your app/ the title of your paper from the list below:
2. Is there another name by which this app is known?
   1. Yes
      1. What is the app called?
   2. No
3. Was the app made available after the end of your study, commercially or otherwise?
   1. Yes
      1. In what geographical locations could the app be accessed? (e.g., in what continents, countries or regions was it accessible?)
      2. Where was it made available?
         1. Apple App Store (iPhone)
         2. Google Play Store
         3. Apple Play Store (Mac)
         4. Non-commercially (e.g., in mental health services, schools, universities)
            1. If you selected 'Non-comercially', in what settings was the app made available?
         5. Other
            1. If you selected Other, please specify:
   2. No
      1. Why was the app not made available after the end of your study?
4. Is the app currently available, commercially, or otherwise?
   1. Yes
      1. In what geographical locations is the app accessible? (e.g., in what continents, countries or regions is it accessible?)
      2. How can it currently be accessed?
         1. Apple App Store (iPhone)
         2. Google Play Store
         3. Apple Play Store (Mac)
         4. Non-commercially (e.g., in mental health services, schools, universities)
            1. If you selected 'Non-comercially', in what settings is the app available?
         5. Other
            1. If you selected Other, please specify:
   2. No
      1. Why is the app not currently available?
      2. Are there any plans to make the app available in the future?
         1. Yes
         2. No
         3. Don't know
         4. Please provide further details:
5. If you have any additional comments about the implementation and sustainability of the app-based intervention described in your study, please include them here:

**Thank you for your responses**

1. Thank you very much for taking the time to complete these questions, we are extremely appreciative of your time. We would like to acknowledge you in any resulting publication and would be grateful if you could confirm that you are happy to be acknowledged in this way.
   1. Yes
   2. No
2. We have an additional five questions that are of interest to us. If you have time, we would be grateful if you could answer these questions for us.
   1. University researchers/academics
   2. Clinicians
   3. Commercial app developers/ tech developers
   4. Young people/ patient and public involvement (PPI)
   5. Other
      1. If you selected Other, please specify:
3. Was the app co-designed with young people?
   1. Yes
      1. In what way was it co-designed?
   2. No
4. How long did it take to develop and test the app? (What was the duration of the research process from inception to the end of the trial?)
5. How was the research funded?
6. How much did it cost to develop and test the app? (Please indicate the currency)

**Thank you very much for your time.**
