# Supplementary material for: Determination of Markers of Successful Implementation of Mental Health Apps for Young People: Systematic Review
Source: J Med Internet Res. 2022 Nov 9;24(11):e40347. doi: 10.2196/40347 (PMC9685513; doi:10.2196/40347)
Supplement: Multimedia Appendix 3 [file jmir_v24i11e40347_app3.docx]

Multimedia Appendix 3. Study characteristics

| Study | Country | App name | Testing an existing app? | Study design | Intervention focus | Population | Sample size, n | Age (years), mean (SD) | Sex (female), % |
| --- | --- | --- | --- | --- | --- | --- | --- | --- | --- |
| Aboody et al [35], 2020 | Israel | GGBI^a^/GG OCD^b^, Anxiety and Depression (body image module) | No | RCT^c^ | None | University students | 90 | 23.51 (1.45) | 100 |
| Bendtsen et al [36], 2020 | Sweden | MHMH | No | RCT | None | University students | 654 | 25^d^ | 78 |
| Borjalilu et al [37], 2019 | Iran | Aramgar | No | Quasi-experimental | Stress | University students | 68 | 24.29 (3.21) | 71 |
| Broglia et al [38], 2019 | United Kingdom | Pacifica/Sanvello | Yes | Feasibility trial | Anxiety and depression | University students | 38 | Intervention: 21(3.24) and TAU^e^: 23 (4.11) | Intervention: 50 and TAU: 67 |
| Bucci et al [39], 2018 | United Kingdom | Actissist | No | Pilot RCT | Early psychosis | Clinical population | 36 | Intervention: 20.21 (7.37) and control: 18.33 (7.0)^f^ | Intervention: 37.5 and control: 75 |
| Cerea et al [40], 2020 | Italy | GG Relationship Doubts (GGRO^g^)/GG OCD—Anxiety and Depression (relationship module) | No | RCT | ROCD^h^ | University students | 50 | 22 (1.32) | 76 |
| Cerea et al [41], 2021 | Italy | GGBI: Positive Body Image/GG OCD—Anxiety and Depression (body image module) | No | RCT | Females at a high risk of developing body image disorders | University students | 50 | 21.74 (1.35) | 100 |
| Di Simplicio et al [42], 2020 | United Kingdom | Imaginator | No | Feasibility trial | Self-harm | University students, school students, general population, and clinical population | 38 | Range 16-25 | 82 |
| Egilsson et al [43], 2021 | Iceland | SidekickHealth | No | Pilot RCT | None | School students | 41 | 15.6 (0.26) | 41 |
| Fish and Saul [44], 2019 | United States | Headspace | Yes | RCT | Depression | University students | 72 | 21 | 94 |
| Fitzpatrick et al [45], 2017 | United States | Woebot | No | RCT | Anxiety and depression | University students | 70 | 22.2 (2.33) | 67 |
| Flett et al [46], 2020 | New Zealand | Headspace | Yes | RCT | None | University students | 250 | 17.87 (0.47) | 67.6 |
| Flett et al [47], 2019 | New Zealand | Headspace and Smiling Mind | Yes | RCT | None | University students | 208 | 20.08 (2.8) | — |
| Franklin et al [48], 2016 | United States | Therapeutic Evaluative Conditioning/TecTec^i^ | No | RCT | Self-injurious thoughts and behaviors | Clinical population | Study 1: 114, study 2: 131, and study 3: 163 | Study 1: 23.02 (5.47), study 2:, 22.91 (4.9), and study 3: 24.50 (6.61) | Study 1: 80.7, study 2: 74.05, and study 3: 58.89 |
| Huberty et al [49], 2019 | United States | Calm | Yes | RCT | Stress | University students | 88 | 20.41 (2.31) | 88 |
| Hur et al [50], 2018 | Korea | Todac | No | RCT | Other specified depressive disorder | Clinical population | 34 | 23.71 (3.26) | 88 |
| Jalal et al [51], 2018 | United Kingdom | Unknown | No | RCT | Healthy individuals with OCD-like contamination fears | General population | 93 | 25.2 (8.0) | 64.5 |
| Kageyama et al [52], 2021 | Japan | SPSRS | No | Pilot RCT | Subthreshold depression | University students | 32 | 20.06 (1.24) | 34.4 |
| Kajitani et al [53], 2020 | Japan | Mental App | No | Quasi-experimental | None | University students | 57 | Intervention: 21.8 (1.60) and control: 21.7 (3.30) | Intervention: 40 and control: 45 |
| Lee and Jung [54], 2018 | Canada | DeStressify | Yes | Pilot RCT | None | University students | 163 | Intervention: 20.3 and control: 20.9 | Intervention: 58 and control: 67 |
| Levin et al [55], 2022 | United States | ACT^j^ Matrix | No | RCT | None | General population | 102 | 23.51 (8.07) | 75 |
| Levin et al [56], 2020 | United States | Stop Breathe Think | Yes | Pilot RCT | Students on waitlist for Counselling and Psychological Services center | University students | 23 | Intervention: 19.9 (2.18) and control: 20.85 (2.67) | 100 |
| Levin et al [57], 2018 | United States | Unknown | No | RCT | Adults high in self-criticism | General population | 87 | 22.76 (7.02) | 69.9 |
| Lyzwinski et al [58], 2019 | Australia | My Student Mindfulness App | No | RCT | None | University students | 72 | 20.19 | 67 |
| McCloud et al [59], 2020 | United Kingdom | Feel Stress Free/Thrive: Mental Wellbeing | Yes | RCT | Anxiety and depression | University students | 168 | 24.3 (6.71) | 82.7 |
| Newman et al [60], 2020 | United States | Lantern | Yes | RCT | Generalized Anxiety Disorder | University students | 100 | Intervention: 21.62 and control: 21.18 | Intervention: 82 and control: 72 |
| O’Dea et al [61], 2020 | Australia | WeClick | No | RCT | Depression and anxiety | General population | 193 | 14.82 (0.94) | 86.5 |
| Orosa-Duarte et al [62], 2021 | Spain | REM Volver a casa^k^ | No | RCT | None | University students | 84 | 23.0 (4.16) | 85 |
| Ponzo et al [63], 2020 | United Kingdom | BioBase | No | RCT | Anxiety and stress | University students | 146 | Intervention: 19.9 (1.83) and control: 19.84 (1.76) | Intervention: 62.5 and control: 63.5 |
| Reid et al [64], 2011 | Australia | Mobiletype | No | RCT | Mild or more mental health concerns | Primary care patients | 114 | Intervention: 18.5 (3.2) and control: 17.4 (3.2) | Intervention: 77.9 and control: 63.0 |
| Rodgers et al [65], 2018 | United States | BodiMojo | No | RCT | None | School students, youth attending youth organizations, and university students | 274 | 18.36 (1.34) | 74 |
| Roncero et al [66], 2019 | Spain | GGRO/GG OCD—Anxiety and Depression (relationship module) | No | RCT | OCD | University students | 97 | iApp: 22.88 (9.23) and dApp: 20.09 (2.73) | iApp: 74.5 and dApp: 89.1 |
| Schlosser et al [67], 2018 | United States | PRIME^l^ | No | RCT | Recent-onset schizophrenia spectrum disorders | Clinical population | 43 | Intervention: 24.32 (2.6) and control: 23.79 (4.5) | Intervention: 40 and control: 35 |
| Yang et al [68], 2018 | United States | Headspace | Yes | RCT | None | University students | 88 | 25.11 | 63.6 |

^a^GGBI: GG Positive Body Image

^b^OCD: obsessive-compulsive disorder.

^c^RCT: randomized controlled trial.

^d^Median.

^e^TAU: treatment as usual.

^f^Age at first symptoms.

^g^GGRO: GG Relationship Doubt & Obsession

^h^ROCD: relationship obsessive-compulsive disorder.

^i^TecTec: Therapeutic Evaluative Conditioning

^j^ACT: acceptance and commitment therapy.

^k^REM Volver a casa: ‘Mindfulness-Based Emotion Regulation. Going Home

^l^PRIME: Personalized Real-time Intervention for Motivational Enhancement.
