# Supplementary material for: Determination of Markers of Successful Implementation of Mental Health Apps for Young People: Systematic Review
Source: J Med Internet Res. 2022 Nov 9;24(11):e40347. doi: 10.2196/40347 (PMC9685513; doi:10.2196/40347)
Supplement: Multimedia Appendix 4 [file jmir_v24i11e40347_app4.docx]

Multimedia Appendix 4. Intervention characteristics

| Study | App | Type of intervention | Aim of intervention | Intervention delivery | Duration | Setting | Gamification | Incentive or reimbursement? |
| --- | --- | --- | --- | --- | --- | --- | --- | --- |
| Aboody et al [35], 2020 | GGBI^a^/GG  OCDb, Anxiety and Depression (body image module) | CBT^c^ based | Promotion | Stand-alone | 2 weeks | University | Yes | Yes |
| Bendtsen et al [36], 2020 | MHMH | Principles of positive psychology | Promotion | Stand-alone | 10 weeks | University | No | Not reported |
| Borjalilu et al [37], 2019 | Aramgar | Emotion-focused coping according to mindfulness-based stress reduction | Promotion | Stand-alone and adjunct | 20 days | University | No | Not reported |
| Broglia et al [38], 2019 | Pacifica/Sanvello | Well-being | Treatment | Adjunct | 6 sessions | University counselling service | No | Not reported |
| Bucci et al [39], 2018 | Actissist | Cognitive therapy | Treatment | Adjunct | 12 weeks | Mental health service(s) | No | Yes |
| Cerea et al [40], 2020 | GG Relationship Doubts (GGRO^d^)/GG OCD—Anxiety and Depression (relationship module) | Challenge dysfunctional beliefs | Treatment | Stand-alone | 15 days | University | Yes | Not reported |
| Cerea et al [41], 2021 | GGBI: Positive Body Image/GG OCD—Anxiety and Depression (body image module) | Cognitive behavioral | Prevention | Stand-alone | 16 days | University | Yes | Not reported |
| Di Simplicio et al [42], 2020 | Imaginator | Functional Imagery Training | Treatment | Adjunct | 8 weeks | Research center, hospital, or charity | Yes | Yes |
| Egilsson et al [43], 2021 | SidekickHealth | Social health game | Promotion | Stand-alone | 6 weeks | School | Yes | Yes |
| Fish and Saul [44], 2019 | Headspace | Gamified mindfulness meditation app | Treatment | Stand-alone | 2 weeks | University | Yes | Not reported |
| Fitzpatrick et al [45], 2017 | Woebot | CBT principles | Treatment | Stand-alone | Up to 20 sessions over 2 weeks | University | Unsure | Yes |
| Flett et al [46], 2020 | Headspace | Mindfulness meditation | Promotion | Stand-alone | 3 months | University | Yes | Yes |
| Flett et al [47], 2019 | Headspace and Smiling Mind | Mindfulness meditation | Promotion | Stand-alone | 10 days | University | Yes | Not reported |
| Franklin et al [48], 2016 | Therapeutic Evaluative Conditioning/TecTec^e^ | Evaluative conditioning—increase aversion to SITB^f^-related stimuli | Treatment | Stand-alone | 1 month | Community | Yes | Yes |
| Huberty et al [49], 2019 | Calm | Mindfulness meditation | Promotion | Stand-alone | 8 weeks | University | Yes | Yes |
| Hur et al [50], 2018 | Todac Todac | Scenario-based CBT | Treatment or reducing symptoms | Stand-alone | 3 weeks | Mental health service(s)+community | No | Not reported |
| Jalal et al [51], 2018 | Unknown | Vicarious exposure | Treatment | Stand-alone | 7 days | Community | No | Not reported |
| Kageyama et al [52], 2021 | SPSRS | Positive word stimuli through videos | Prevention | Stand-alone | 5 weeks | University | No | Not reported |
| Kajitani et al [53], 2020 | Mental App | Self-monitoring, self-screening, and referral | Prevention | Stand-alone | 2 weeks | University | No | Not reported |
| Lee and Jung [54], 2018 | DeStressify | Mindfulness-based app | Prevention | Stand-alone | 4 weeks | University | No | Yes |
| Levin et al [55], 2022 | ACT^g^ Matrix | Self-monitoring physical activity and dietary behavior based on ACT | Promotion | Stand-alone | 4 weeks | University | Not reported | Yes |
| Levin et al [56], 2020 | Stop Breathe Think | Mindfulness meditation | Treatment | Stand-alone | 4 weeks | University counselling service | Not reported | Not reported |
| Levin et al [57], 2018 | Unknown | Cognitive defusion or restructuring | Promotion | Stand-alone | 2 weeks | University and community | Not reported | Not reported |
| Lyzwinski et al [58], 2019 | My Student Mindfulness App | Student-tailored mindfulness app for weight, weight-related behaviors, and stress | Prevention | Stand-alone | 11 weeks | University | No | Yes |
| McCloud et al [59], 2020 | Feel Stress Free/Thrive: Mental Wellbeing | CBT | Treatment | Stand-alone | 6 weeks | University | Yes | No |
| Newman et al [60], 2020 | Lantern | Guided self-help for GAD^h^ | Treatment | Stand-alone | 3 months | University | No | Yes |
| O’Dea et al [61], 2020 | WeClick | Relationship-focused interactive story-telling app | Promotion | Stand-alone | 4 weeks | Home | No | Yes |
| Orosa-Duarte et al [62], 2021 | REM^i^ Volver a casa | Digital mindfulness training app | Promotion | Stand-alone | 8 weeks | University | No | Yes |
| Ponzo et al [63], 2020 | BioBase | Psychoeducational | Promotion | Stand-alone | 4 weeks | University | No | Yes |
| Reid et al [64], 2011 | Mobiletype | Mental health assessment and management | Treatment | Adjunct | 2-4 weeks | Primary care | No | Yes |
| Rodgers et al [65], 2018 | BodiMojo | Promotes positive body image through self-compassion | Promotion | Stand-alone | 6 weeks | University, secondary school, and youth organization | No | Yes |
| Roncero et al [66], 2019 | GGRO/GG OCD—Anxiety and Depression (relationship module) | A novel cognitive training app (GGRO) designed to challenge OCD-related beliefs | Treatment | Stand-alone | 15 days | University | Yes | Yes |
| Schlosser et al [67], 2018 | PRIME^j^ | Includes a peer community, goal and achievement tracking, and cognitive CBT-based coaching | Treatment | Stand-alone | 12 weeks | Home | No | Yes |
| Yang et al [68], 2018 | Headspace | Audio-guided mindfulness meditation | Promotion | Stand-alone | 30 days | University | No | Not reported |

^a^GGBI: GG Positive Body Image

^b^OCD: obsessive-compulsive disorder.

^c^CBT: cognitive behavioral therapy.

^d^GGRO: GG Relationship Doubt & Obsession

^e^TecTec: Therapeutic Evaluative Conditioning

^f^SITB: self-injurious thoughts and behaviors.

^g^ACT: acceptance and commitment therapy.

^h^GAD: Generalized Anxiety Disorder.

^i^REM Volver a casa: ‘Mindfulness-Based Emotion Regulation. Going Home

^j^PRIME: Personalized Real-time Intervention for Motivational Enhancement.
