# Supplementary material for: Determination of Markers of Successful Implementation of Mental Health Apps for Young People: Systematic Review
Source: J Med Internet Res. 2022 Nov 9;24(11):e40347. doi: 10.2196/40347 (PMC9685513; doi:10.2196/40347)
Supplement: Multimedia Appendix 5 [file jmir_v24i11e40347_app5.docx]

Multimedia Appendix 5. Implementation and sustainability.

| Study | App | Testing an existing app? | Was the app made available after the end of the study? | Is the app currently available? | In what geographical location could or can it be accessed? | How can it currently be accessed? | Are there any plans to make the app available in the future? | Was the app co-designed with young people? | How long did it take to develop and test the app? |
| --- | --- | --- | --- | --- | --- | --- | --- | --- | --- |
| Aboody et al [35], 2020 | GGBI^a^/GG  OCD^b^, Anxiety and Depression (body image module) | No | Yes | Yes | Worldwide | Apple App Store (iPhone) and Google Play Store | Yes | No | Not reported |
| Bendtsen et al [36], 2020 | MHMH | No | Yes | Yes | Sweden | Universities and noncommercial websites | Do not know | No | 6 months |
| Borjalilu et al [37], 2019 | Aramgar | No | Yes | Yes | Iran | Mental health services | Yes | No | >1 year |
| Broglia et al [38], 2019 | Pacifica/Sanvello | Yes | Yes | Yes | United States and United Kingdom (as well as others) | Apple App Store (iPhone or Mac) and Google Play Store | Do not know | No | Do not know |
| Bucci et al [39], 2018 | Actissist | No | No | No | — | — | Yes | Yes | 2 trials: 6 years |
| Cerea et al [40], 2020 | GG Relationship Doubts (GGRO^c^)/GG OCD—Anxiety and Depression (relationship module) | No | Yes | Yes | Worldwide | Apple App Store (iPhone) and Google Play Store | Yes | No | — |
| Cerea et al [41], 2021 | GGBI: Positive Body Image/GG OCD—Anxiety and Depression (body image module) | No | Yes | Yes | Worldwide | Apple App Store (iPhone) and Google Play Store | Yes | No | — |
| Di Simplicio et al [42], 2020 | Imaginator | No | Yes | Yes | England | CAMHS^d^ | Yes | Yes | 6 months |
| Egilsson et al [43], 2021 | SidekickHealth | No | Yes | Yes | Worldwide | Apple App Store (iPhone) and Google Play Store | Yes | Yes | 4 years |
| Franklin et al [48], 2016 | Therapeutic Evaluative Conditioning/TecTec^e^ | No | Yes | No | Do not know | Apple App Store (iPhone or Mac) and Google Play Store | Do not know | No | 2 years |
| Hur et al [50], 2018 | Todac Todac | No | Yes | Yes | South Korea | Apple App Store (iPhone) and Google Play Store | Yes | No | 2 years |
| Jalal et al [51], 2018 | Unknown | No | No | No | — | — | Do not know | No | 2-3 years |
| Lee and Jung [54], 2018 | DeStressify | Yes | Yes | Yes | Worldwide | Apple App Store (iPhone) and Google Play Store | Do not know | Yes | — |
| Levin et al [55], 2022 | ACT^f^ Matrix | No | No | No | — | — | No | No | Development 2 weeks, testing 1 year |
| Levin et al [57], 2018 | Unknown | No | No | No | — | — | No | No | Development 2 weeks, testing 1 year |
| Lyzwinski et al [58], 2019 | My Student Mindfulness App | No | No | No | — | — | Do not know | Not reported | 1 year |
| McCloud et al [59], 2020 | Feel Stress Free/Thrive: Mental Wellbeing | Yes | Yes | Yes | United Kingdom (maybe more) | Apple App Store (iPhone), Google Play Store, and noncommercially (eg, in mental health services, schools, universities) | Yes | No | — |
| Newman et al [60], 2020 | Lantern | Yes | No | No | — | — | No | Yes | A few years |
| O’Dea et al [61], 2020 | WeClick | No | Yes | No | Australia | Was available via a web-based mental health service | Yes | Yes | A few years |
| Ponzo et al [63], 2020 | BioBase | No | No | Yes | United Kingdom and United States | Industrial deployment (well-being services in corporate organizations) | Yes | No | 2 years |
| Rodgers et al [65], 2018 | BodiMojo | No | Do not know | No | — | — | No | Yes | 18 months |
| Roncero et al [66], 2019 | GGRO/GG OCD—Anxiety and Depression (relationship module) | No | Yes | Yes | Worldwide | Apple App Store (iPhone) and Google Play Store | Yes | No | — |
| Schlosser et al [67], 2018 | PRIME^g^ | No | Yes | No | Not reported | Via additional clinical studies that allowed for global access, but contingent on meeting the eligibility criteria | Yes | Yes | 12-18 months |

^a^GGBI: GG positive body image

^b^OCD: obsessive-compulsive disorder.

^c^GGRO: GG Relationship Doubt & Obsession

^d^CAMHS: Child and adolescent mental health services

^e^TecTec: Therapeutic Evaluative Conditioning

^f^ACT: acceptance and commitment therapy.

^g^PRIME: Personalized Real-time Intervention for Motivational Enhancement.
