# Supplementary material for: Determination of Markers of Successful Implementation of Mental Health Apps for Young People: Systematic Review
Source: J Med Internet Res. 2022 Nov 9;24(11):e40347. doi: 10.2196/40347 (PMC9685513; doi:10.2196/40347)
Supplement: Multimedia Appendix 6 [file jmir_v24i11e40347_app6.docx]

Multimedia Appendix 6. Implementation variables.

| Study | Coproduction | Acceptability | Adoption | Appropriateness | Feasibility | Fidelity | Implementation cost | Engagement | Penetration | Sustainability |
| --- | --- | --- | --- | --- | --- | --- | --- | --- | --- | --- |
| Aboody et al [35], 2020 | Not coproduced | Not reported | Available in the app marketplace | Not reported | Not reported | Not reported | Not reported | Adherence and attrition measured | Not reported | Not reported |
| Bendtsen et al [36], 2020 | Not coproduced | Not reported | Available in universities and noncommercial websites | Not reported | Not reported | Not reported | Not reported | Attrition reported. Primary outcome data were collected from 61.2% participants in the intervention | Not reported | Not reported |
| Borjalilu et al [37], 2019 | Not coproduced | Not reported | Available in mental health services and universities | Not reported | Not reported | Not reported | Not reported | Not reported | Not reported | Not reported |
| Broglia et al [38], 2019 | Not coproduced | Randomization acceptability assessed | Evaluation of existing consumer app available in the app marketplace | Not reported | Reported | Reported | Not reported | Study retention measured | Not reported | Not reported |
| Bucci et al [39], 2018 | Coproduced | Assessed via participant feedback | In the process of ORCHA^a^ review, data analysis and then release | Assessed via participant feedback | Assessed using uptake, completion, and data entries | Not reported | Not reported | Retention and data entries reported | Not reported | Not reported |
| Cerea et al [40], 2020 | Not coproduced | Not reported | Available in the app marketplace | Not reported | Not reported | Not reported | Not reported | Adherence and attrition measured | Not reported | Not reported |
| Cerea et al [41], 2021 | Not coproduced | Not reported | Available in the app marketplace | Not reported | Not reported | Not reported | Not reported | Adherence and attrition measured | Not reported | Not reported |
| Di Simplicio et al [42], 2020 | Coproduced | Not reported | Made available to child and adolescent mental health practitioners | Not reported | Average number of sessions completed by participants, intervention completion, and follow-up assessment completion | Not reported | Not reported | Average number of sessions attended | Not reported | Not reported |
| Egilsson et al [43], 2021 | Coproduced | Assessed using the Systematic Usability Scale | Available in the app marketplace | Not reported | Measured in-app exercise completion | Not reported | Not reported | Completion of in-app exercises | Not reported | Not reported |
| Fish and Saul [44], 2019 | Not reported | Not reported | Evaluation of existing consumer app available in the app marketplace | Not reported | Not reported | Not reported | Not reported | Adherence and attrition measured | Not reported | Not reported |
| Fitzpatrick et al [45], 2017 | Not reported | Assessed using mixed-format user feedback | Available in the app marketplace | Assessed using mixed-format user feedback | Assessed using mixed-format user feedback | Not reported | Not reported | Recorded total number of interactions with the bot and attrition | Not reported | Not reported |
| Flett et al [46], 2020 | Not reported | Assessed using 2 Likert scale questions | Evaluation of existing consumer app available in the app marketplace | Assessed using 2 Likert scale questions | Not reported | Not reported | Not reported | Completed meditation sessions, time spent meditating, and study attrition measured | Not reported | Not reported |
| Flett et al [47], 2019 | Not reported | Assessed using 2 Likert scale questions | Evaluation of existing consumer app available in the app marketplace | Assessed using 2 Likert scale questions | Not reported | Not reported | Not reported | App adherence and study attrition measured | Not reported | Not reported |
| Franklin et al [48], 2016 | Not coproduced | Not reported | Not adopted | Not reported | Not reported | Not reported | Not reported | App use and study retention measured | Not reported | Not reported |
| Huberty et al [49], 2019 | Not reported | Acceptability measured with a satisfaction survey | Evaluation of existing consumer app available in the app marketplace | Intent to continue use measured | Feasibility measures included acceptability and demand | Not reported | Not reported | Adherence and study retention measured | Not reported | Not reported |
| Hur et al [50], 2018 | Not coproduced | Not reported | Available in the app marketplace | Not reported | Not reported | Not reported | Not reported | Study retention measured | Not reported | Not reported |
| Jalal et al [51], 2018 | Not coproduced | Not reported | Not adopted | Not reported | Not reported | Not reported | Not reported | Adherence measured | Not reported | Not reported |
| Kageyama et al [52], 2021 | Not reported | Not reported | Not adopted | Not reported | Not reported | Not reported | Not reported | Adherence measured | Not reported | Not reported |
| Kajitani et al [53], 2020 | Coproduced with students | User satisfaction measured | Not adopted | Intent to continue use measured | Not reported | Not reported | Not reported | App use data collected and study retention measured | Not reported | Not reported |
| Lee and Jung [54], 2018 | Coproduced through market research and beta testing | Not reported | Evaluation of existing consumer app no longer available in the app marketplace | Not reported | Not reported | Not reported | Not reported | Self-reported app use and adherence | Not reported | Not reported |
| Levin et al [55], 2022 | Not coproduced | SUS^b^ measured usability and acceptability | Not adopted | Not reported | Not reported | Not reported | Not reported | Self-reported app use and study retention | Not reported | Not reported |
| Levin et al [56], 2020 | Not reported | SUS measured usability and acceptability | Evaluation of existing consumer app available in the app marketplace | Survey measured helpfulness, ease of use, and perceived fit | Assessed via response rates | Not reported | Not reported | Self-reported app use and study retention | Not reported | Not reported |
| Levin et al [57], 2018 | Not reported | SUS measured usability and acceptability | Not adopted | Not reported | Not reported | Not reported | Not reported | Self-reported app use and study retention | Not reported | Not reported |
| Lyzwinski et al [58], 2019 | Not reported | Survey assessed likabilityand cceptability | Not adopted | Not reported | Feasibility was assessed on the basis of participant retention and adherence | Not reported | Not reported | Retention and adherence measured | Not reported | Not reported |
| McCloud et al [59], 2020 | Not coproduced | Not reported | Evaluation of existing consumer app available in the app marketplace | Not reported | Not reported | Not reported | Not reported | Self-reported use, attrition, and adherence measured | Not reported | Not reported |
| Newman et al [60], 2020 | Coproduced | Not reported | Evaluation of existing consumer app no longer available in the app marketplace | Not reported | Not reported | Not reported | Not reported | App use and study retention measured | Not reported | Not reported |
| O’Dea et al [61], 2020 | Coproduced | Satisfaction and usefulness questionnaire | Initially made available via a web-based mental health service to participating schools but no longer available | Satisfaction and usefulness questionnaire | Not reported | Not reported | Not reported | App use and study retention measured | Not reported | Not reported |
| Orosa-Duarte et al [62], 2021 | Not reported | Not reported | Available in the app marketplace | Not reported | Not reported | Not reported | Not reported | Study retention | Not reported | Not reported |
| Ponzo et al [63], 2020 | Not coproduced | Feedback questionnaire with both multiple-choice questions and free-text answers | Available noncommercially—industrial deployment | Feedback questionnaire with both multiple-choice questions and free-text answers | Feedback questionnaire with both multiple-choice questions and free-text answers | Not reported | Not reported | App use and study retention measured | Not reported | Not reported |
| Reid et al [64], 2011 | Not reported | Not reported | Not reported | Not reported | Not reported | Not reported | Not reported | App use and study retention | Not reported | Not reported |
| Rodgers et al [65], 2018 | Coproduced | Satisfaction, experience, and ratings of features measured | Not adopted | Intent to continue use measured | Feedback questionnaire and interviews conducted | Not reported | Not reported | App use and study retention | Not reported | Not reported |
| Roncero et al [66], 2019 | Not coproduced | Not reported | Available in the app marketplace | Not reported | Not reported | Not reported | Not reported | Adherence and attrition measured | Not reported | Not reported |
| Schlosser et al [67], 2018 | Coproduced with key stakeholders | Assessed using an exit interview | Not yet adopted | Assessed using an exit interview | Feasibility assessed via use data (log-in frequency, challenge completion, and interactions) | Not reported | Not reported | Study retention and app use measured | Not reported | Not reported |
| Yang et al [68], 2018 | Not reported | Not reported | Evaluation of existing consumer app available in the app marketplace | Not reported | Feasibility assessed by continued use over 30 days | Not reported | Not reported | Retention | Not reported | Not reported |

^a^ORCHA: Organisation for the Review of Care and Health Apps

^b^SUS: Systematic Usability Scale.
